# Adverse events after manual therapy among patients seeking care for neck and/or back pain: a randomized controlled trial

**DOI:** 10.1186/1471-2474-15-77

**Published:** 2014-03-12

**Authors:** Kari Paanalahti, Lena W Holm, Margareta Nordin, Martin Asker, Jessica Lyander, Eva Skillgate

**Affiliations:** 1 Institute of Environmental Medicine, Karolinska Institutet, Box 210, SE-17177 Stockholm, Sweden; 2 Occupational and Industrial Orthopaedic Center (OIOC), NYU Hospital for Joint Diseases, New York University Langone Medical Center, 63 Downing Street, New York, NY 10014, USA; 3 Scandinavian College of Naprapathic Manual Medicine, Kräftriket 23A, SE-11419 Stockholm, Sweden

**Keywords:** Naprapathy, Manual therapy, Adverse event, RCT

## Abstract

**Background:**

The safety of the manual treatment techniques such as spinal manipulation has been discussed and there is a need for more information about potential adverse events after manual therapy. The aim of this randomized controlled trial was to investigate differences in occurrence of adverse events between three different combinations of manual treatment techniques used by manual therapists (i.e. chiropractors, naprapaths, osteopaths, physicians and physiotherapists) for patients seeking care for back and/or neck pain. In addition women and men were compared regarding the occurrence of adverse events.

**Methods:**

Participants were recruited among patients, ages 18–65, seeking care at the educational clinic of the Scandinavian College of Naprapathic Manual Medicine in Stockholm. The patients (n = 767) were randomized to one of three treatment arms 1) manual therapy (i.e. spinal manipulation, spinal mobilization, stretching and massage) (n = 249), 2) manual therapy excluding spinal manipulation (n = 258) and 3) manual therapy excluding stretching (n = 260). Treatments were provided by students in the seventh semester of total eight. Adverse events were measured with a questionnaire after each return visit and categorized in to five levels; 1) short minor, 2) long minor, 3) short moderate, 4) long moderate and 5) serious adverse events, based on the duration and/or severity of the event. Generalized estimating equations were used to examine the association between adverse event and treatments arms.

**Results:**

The most common adverse events were soreness in muscles, increased pain and stiffness. No differences were found between the treatment arms concerning the occurrence of adverse event. Fifty-one percent of patients, who received at least three treatments, experienced at least one adverse event after one or more visits. Women more often had short moderate adverse events (OR = 2.19 (95% CI: 1.52-3.15)), and long moderate adverse events (OR = 2.49 (95% CI: 1.77-3.52)) compared to men.

**Conclusion:**

Adverse events after manual therapy are common and transient. Excluding spinal manipulation or stretching do not affect the occurrence of adverse events. The most common adverse event is soreness in the muscles. Women reports more adverse events than men.

**Trial registration:**

This trial was registered in a public registry (Current Controlled Trials) (ISRCTN92249294).

## Background

The lifetime prevalence of neck and/or back pain is up to 80% [1,2]. Manual therapies are commonly used to treat these conditions and some countries recommend spinal manipulation as a treatment option in their clinical guidelines [3]. The safety of manual treatment techniques has been discussed and the main concern being raised is the potential harmful consequences following spinal manipulation in the upper neck region [4,5]. Most studies show that unwanted adverse events due to manual treatments, like spinal manipulation are common, but the severity and duration of such events are mild and transient [5-8]. Several studies have failed to demonstrate that spinal manipulation causes cerebrovascular lesion [9-11]. Instead, it has been suggested that the associations between cervical manipulation and cerebrovascular lesions seen in one study, is likely to be a result of reversed causality [9]. However, we cannot rule out the possibility, that cervical manipulation in extremely rare cases, may cause a serious adverse event [7]. Previous studies have suggested that the treatment effect when comparing spinal manipulation with spinal mobilization seems to be equal but the presence of adverse events appears to be more common among patients treated with spinal manipulation [12,13]. Stretching has been demonstrated to be an effective treatment method for neck pain [14] but others has suggested that the effect for musculoskeletal pain as well as the role of stretching for the occurrence of adverse events are uncertain [15]. Further, it has been suggested that women experience more adverse events after manual treatments than men [16].

Spinal manipulation, spinal mobilization and stretching are used as treatment methods by different professions within manual medicine such as chiropractors, naprapaths, osteopaths, physicians and physiotherapists.

Naprapathy in Scandinavia is defined as a system for specific examination, diagnostics, manual treatment and rehabilitation of pain and dysfunction in the musculoskeletal system. Manual therapy provided by naprapaths is called Soft and Connective Tissue Manipulations (SCTM), and is a combination of manual techniques as spinal manipulation/mobilization, stretching and massage used to treat shortened or pathologic soft and connective tissue that are thought to be common causes to these pain conditions [17]. The profession of naprapathy was initiated in 1907 in the United States, and is today practiced mainly in Sweden, Finland, Norway and the United States. Naprapath is a registered health profession in Sweden since 1994 and is controlled by The National Board of Health and Welfare. Research about naprapathy is requested in Scandinavia.

The occurrence of adverse events after manual therapy provided by naprapaths has been reported in one previous study [17]. However, the focus of that study was not to explore the occurrence of adverse events and consequently more research is needed on this topic.

The overall aim of this study was to describe the occurrence and severity of adverse events after manual therapy for patients seeking care for neck and/or back pain. The specific aim was to investigate differences in the occurrence of adverse events of varying severity and duration, between a) manual therapy (a combination of spinal manipulation, spinal mobilization, muscle stretching and/or massage), b) manual therapy without spinal manipulation and c) manual therapy without muscle stretching. An additional aim was to compare men and women regarding the occurrence of adverse events.

## Methods

### Design

The study is a three-arm randomized controlled trial (RCT) called the Stockholm MINT-trial (the Stockholm Manual Intervention Trial).

The trial was approved by the Ethical review board in Stockholm, Sweden, 2009/1848-31/2, and the trial was registered in a public registry (Current Controlled Trials (ISRCTN92249294)).

This report is based on the trial registered in the Current Controlled Trials with the primary objective to report on the treatment effects, of different combinations of manual therapy for patients seeking care for neck and/or back pain with one-year follow-up. The secondary objective was to describe the occurrence and severity of adverse events directly after manual therapy for patients seeking care for neck and/or back pain. This article reports adverse events from the RCT.

### Setting

The RCT was conducted at the educational clinic of the Scandinavian College of Naprapathic Manual Medicine, Stockholm, Sweden. The education program to become a naprapath is four years of full time studies. The students, who provided the treatments in this trial are in their 7th semester, and they have regularly treated patients during five semesters, including spinal mobilization techniques since three semesters and spinal manipulation techniques since two semesters under supervision of experienced registered naprapaths. They have also passed all practical clinical examinations at this level of the education.

### Participants

#### *Inclusion criteria*

The participants eligible to this trial were patients, 18–65 years old, seeking care for neck and/or back pain and had not visited the educational clinic during the previous month.

#### *Exclusion criteria*

Participants were not included if any of the following criteria were present: (1) not mastering the Swedish language, (2) having scored pain <2 in two questions regarding pain (pain at the present time and the worst pain during the past four weeks) in neck and/or back on a numerical rating scale (0–10), (3) pregnancy, (4) current or past cancer, (5) having received treatments for the current complaint by a chiropractor, naprapath, osteopath or physiotherapist during the past month, (6) duration of the current complaint less than one week, (7) requiring/refusing spinal manipulation/stretching, (8) contraindication for spinal manipulation according to the Swedish Board of Social Welfare [18], (9) no indication for spinal manipulation in the area of complaint, (10) red flags (for example previous trauma, inflammatory or rheumatic diseases, drug addict, large rapid weight decrease etc.), (11) specific diagnosis (for example ankylosing spondylitis, spinal stenosis, rheumatoid arthritis), (12) sick leave due to planned/completed surgery for neck and/or back.

A trained receptionist marked all potential study participants in a ledger with a specific color as the first step according to inclusion criteria; age, neck and/or back pain and no visit at the educational clinic during the past month. The following steps in the process were handled by the supervised student therapists: trial information, informed consent, the baseline data collection, the decision about inclusion, and the randomization allocation. The supervised student therapists as well as the supervising experienced registered naprapaths were thoroughly trained in all aspects of the study protocol at several meetings before the start of the study. Ambiguities and questions were regularly discussed in meetings during the inclusion period.

### Randomization

The randomization was carried out in advance by a trained research assistant. The assistant prepared sequentially numbered opaque and sealed envelopes with cards numbered 1, 2 or 3, manually randomized by drawing these cards from a box.

We used stratified randomization based on the location of pain: (1) neck and upper back (including neck/shoulders and upper back, above the 11th thoracic vertebra, upper extremities and chest), (2) lower back (including the area below the 10th thoracic vertebra, gluteal area and lower extremities) and (3) neck and back (pain equally bad in neck and upper back and lower back). For each stratum, randomization with blocking was used so that in the end of each block of 99 patients there were equal allocations of treatment to three treatment arms.

At the first visit all potential study participants were informed about the study, the meaning of involvement, that participation was voluntary and that they could withdraw at any time without consequences for the treatment. Informed consent was obtained and patients completed the baseline questionnaire suited for the area of pain before randomization. The questionnaires were integrated with serial numbers unique for each patient. The process was then followed by physical examinations and diagnostic assessment by the student therapists. To keep the patient and the therapist unaware to the group assignment until after all baseline data were collected, the unmasking of treatment allocation arm was performed by the therapist after the physical examination and the completing of the baseline questionnaire. The therapists were told not to tell the result of the randomization to the patient if possible, but there was no guarantee for that the patients were blinded to the treatment. Each visit was scheduled for 45 minutes for all treatment arms.

### Intervention

1. Manual therapy: The therapist was allowed to use all manual treatment techniques i.e. spinal manipulation, spinal mobilization, muscle stretching and massage.

2. Manual therapy excluding spinal manipulation: The therapist was allowed to use all manual treatment techniques except for spinal manipulation.

3. Manual therapy excluding muscle stretching: The therapist was allowed to use all manual treatment techniques except for muscle stretching.

The study design was pragmatic and the therapist decided how to combine the allowed manual techniques for each patient within each treatment arm including contra indications. To ensure that the treatments were performed according to the randomization protocol, control of a random number (6%) of medical records was performed post treatment.

### Baseline questionnaire

The baseline questionnaire is based on questions used in previous studies [17,19] and it includes socio-demographic factors, physical activity, smoking habits, previous pain conditions concerning the current complaint and how the current complaint began, previous naprapath treatments and the expectation of recovery and general health [20]. Further, to assess pain and disability a modified Chronic Pain Questionnaire (CPQ) developed by von Korff was used [21], the original scale that was based on recall of the past 6 months was changed to the past 4 weeks [21].

### Follow-up and clinical outcomes

Information of adverse events was collected by a questionnaire completed by the patient in the waiting room before the scheduled treatment at each return visit (maximum six visits within six weeks). If a patient did not show up at the scheduled return visit, the therapist contacted the patient and made a new appointment. After the last visit or in a case of incomplete answers in the questionnaire, a trained research assistant contacted the patient by phone, mail or letter and completed the missing answers, at least three times before the patient was dropped.

The adverse events of concern were events that had occurred within 24 hours following the treatment [16,22,23]. The adverse event questionnaire contained the questions; (1) if the patient had experienced an event as an effect of the treatment given at the latest visit (yes/no), (2) how many hours the event lasted (duration) and (3) to what extent the event had bothered the patient, measured by an 11-point numerical rating scale (NRS) from 0–10 (0 = had not bothered them at all and 10 = had bothered them in the worst possible way).

The choice of adverse events to include in the questionnaire was based on information and results from previous studies [6,7,17,24]; (1) tiredness, (2) soreness in muscles, (3) stiffness,(4) increased pain, (5) nausea, (6) headache, (7) dizziness or (8) “other”. For the data presentation and analyses, the outcome adverse events were categorized into five levels with definitions based on duration and/or severity of the reaction: 1) Short minor (NRS ≤ 3 and < 24 hours of duration), 2) Long minor (NRS ≤ 3 and ≥ 24 hours of duration), 3) Short moderate (NRS > 3 and < 24 hours of duration), 4) Long moderate (NRS > 3 and ≥ 24 hours of duration) and 5) Serious adverse event (the patient had a loss of bowel/bladder function, stroke, fracture or where hospitalized).

### Statistical methods

Descriptive statistics were used to summarize baseline characteristics and describe the frequency and proportion of different types of adverse events after each visit. Odds ratio with 95% confidence intervals were calculated with Generalized Estimating Equations (GEE) to examine the association between adverse events (four levels) and treatment (three arms) and sex respectively with the longitudinal data [25,26]. Potential confounding factors considered were the factors reported in Table 1. No confounding was identified. An additional analysis was done for the number and proportion of patients who had experienced any kind of adverse events, regardless of severity and duration, after every visit, after any of the visits or who had no adverse events after any of the visits were described among patients who had at least three treatments.

**Table 1:** Baseline characteristics of the patients stratified for treatment arms (n = 767)

	**MT**^**a**^	**MT excluding spinal manipulation**	**MT excluding stretching**
	**n = 249**	**n = 258**	**n = 260**
Mean age	35.0	35.3	35.7
(SD)	(12.4)	(12.3)	(11.3)
Women, %	67	74	75
Painful area, %			
Back	35	33	33
Neck	54	54	54
Back/Neck	11	13	13
Duration of the pain, %			
1 week	17	17	18
2–4 weeks	28	23	25
1–3 months	17	21	20
3–6 months	9	10	6
>6 months	29	29	31
^b^Pain at baseline	5.5	5.3	5.5
(SD)	(1.7)	(1.7)	(1.8)
^c^Disability at baseline	2.5	2.5	2.6
(SD)	(2.2)	(2.3)	(2.2)
Education, %			
1–9 years	3	5	3
10–12	40	37	36
13–15	46	46	47
>16	11	12	14
General health, %			
Excellent	16	15	18
Very good	46	48	43
Good	31	30	35
Somewhat	6	7	3
Bad	1	0	1
Daily smoking, %	18	14	17

^a^Manual Therapy (spinal manipulation/mobilization, stretching and massage).

^b^Pain at baseline was based on three pain items: current pain, worst pain, average pain during the past four weeks, measured with a numeric rating scale, 0–10 (0 = no pain, 10 = pain as bad it could be) and calculated as an average pain score for these items.

^c^Disability at baseline was based on three disability items: interference with daily activities, recreational and social activities, and work activities, measured with numeric rating scale, 0–10 (0 = no interference, 10 = unable to carry on with these activities) and calculated as an average disability score for these items.

The data analyses were performed using STATA 12.0 [27].

### Data quality assurance and monitoring

Raw data input from the original questionnaires was made by a trained research assistant. The validity of the data input was tested at least once for every tenth questionnaire, comparing the information with the original baseline- and adverse event questionnaires to the registered data in the data base.

## Results

**Figure 1:**
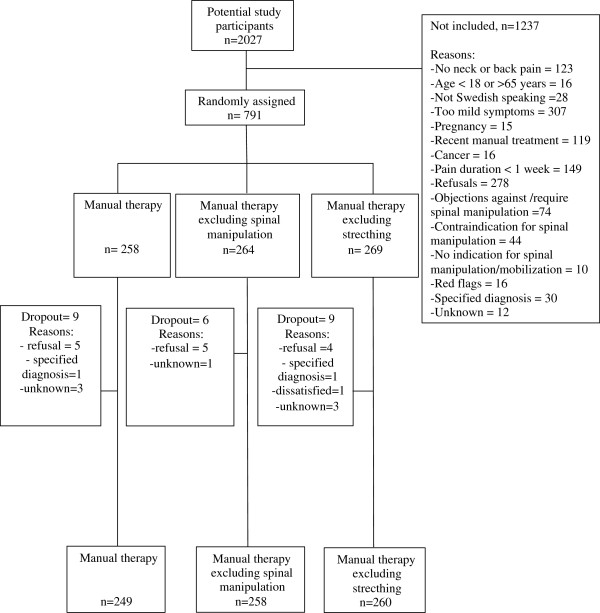
Flowchart of the recruitment and randomization.

Of the 2027 eligible study patients, 1236 did not fulfill the inclusions criteria, and accordingly 791 study participants were randomly assigned to one of the three arms in this study. A total of 24 patients dropped out after randomization (14 wanted to leave the study, two had specific diagnoses, one was dissatisfied and seven had unknown reasons). Therefor the study population was 767 patients (Figure 1). This gives a participating rate of 97%. We had an additional 6% lost to follow-up (of these six percent data was not available regarding first treatment 29%, last treatment, 51%, or any treatment 20%). This missing data was equally distributed between the three treatment arms. Table 1 shows the baseline characteristics of the patients in the trial. Most of the patients were women (72%) and the mean age was 35.0 (SD 12.1) years. The most common pain location was the neck (54%). The proportion of patients with pain for more than six months was 29%. Nearly two thirds of the patients rated their general health to be very good or excellent (Table 1). The randomization and raw data input was made by a trained research assistant not involved in the analyses or in the writing of the manuscript. This person also performed the validation of the data input.

At baseline, the mean pain was 5.4/10 (SD 1.7) and the mean disability was 2.5/10 (SD 2.2) (Table 1), measured with numeric rating scale (0–10). The total numbers of visits were 2692 (mean 3.5 per patient). The number of therapist was 260 and they included patients during January 25th 2010 - December 2nd 2010 and August 26th 2011 - December 3rd 2011.

Table 2 present the number and proportion of patients who reported different types and levels of adverse event after each treatment visit. The most common adverse event was soreness in the muscles followed by increased pain, stiffness and tiredness. No serious adverse events were reported.

**Table 2:** The number and proportions of specific types and levels of adverse events after each visit

**Visit number**	**1 (n = 767)**	**2 (n = 685)**	**3 (n = 556)**	**4 (n = 389)**	**5 (n = 211)**	**6 (n = 84)**
**Type and level of the side-effect**	**n (%)**	**n (%)**	**n (%)**	**n (%)**	**n (%)**	**n (%)**
**Soreness**						
Any level^a^	341 (44)	353 (51)	249 (44)	154 (39)	90 (43)	41 (49)
Short minor	199 (26)	124 (18)	90 (16)	64 (16)	36 (17)	23 (27)
Long minor	0	85 (12)	54 (10)	28 (7)	25 (12)	9 (11)
Short moderate	54 (7)	61 (9)	52 (9)	29 (7)	18 (9)	4 (5)
Long moderate	88 (11)	83 (12)	53 (9)	33 (9)	11 (5)	5 (6)
**Pain**						
Any level	145 (19)	140 (20)	74 (13)	47 (12)	23 (11)	13 (15)
Short minor	27 (4)	23 (3)	18 (3)	10 (2)	4 (2)	3 (4)
Long minor	13 (2)	16 (2)	8 (1)	8 (2)	8 (4)	3 (4)
Short moderate	40 (5)	47 (7)	13 (2)	10 (2)	5 (2)	3 (4)
Long moderate	65 (8)	54 (8)	35 (6)	19 (5)	6 (3)	4 (5)
**Stiffness**						
Any level	108 (14)	105 (15)	61 (11)	36 (9)	25 (11)	6 (7)
Short minor	29 (4)	23 (3)	15 (3)	8 (2)	5 (2)	2 (2)
Long minor	16 (2)	23 (3)	13 (2)	11 (3)	11 (5)	1 (1)
Short moderate	24 (3)	24 (4)	15 (3)	8 (2)	6 (3)	0
Long moderate	39 (5)	35 (5)	18 (3)	9 (2)	3 (1)	3 (4)
**Tiredness**						
Any level	100 (12)	134 (20)	81 (14)	52 (13)	21 (10)	12 (14)
Short minor	55 (7)	74 (11)	39 (7)	32 (8)	12 (6)	7 (8)
Long minor	3 (0)	4 (1)	6 (1)	1 (0)	1 (0)	0
Short moderate	31 (4)	43 (6)	29 (5)	10 (3)	7 (3)	4 (5)
Long moderate	11 (1)	13 (2)	7 (1)	9 (2)	1 (1)	1 (1)
**Headache**						
Any level	83 (11)	104 (15)	68 (12)	34 (8)	12 (6)	8 (10)
Short minor	23 (3)	32 (5)	25 (4)	5 (1)	6 (3)	3 (4)
Long minor	4 (1)	10 (1)	4 (1)	5 (1)	0	0
Short moderate	40 (5)	41 (6)	24 (4)	16 (4)	4 (2)	4 (5)
Long moderate	16 (2)	21 (3)	15 (3)	8 (2)	2 (1)	1 (1)
**Dizziness**						
Any level	79 (11)	53 (8)	33 (6)	14 (4)	9 (4)	4 (4)
Short minor	28 (4)	31 (4)	17 (3)	7 (2)	6 (3)	1 (1)
Long minor	27 (4)	2 (0)	0	0	0	0
Short moderate	10 (1)	13 (2)	14 (3)	6 (2)	3 (1)	2 (2)
Long moderate	14 (2)	7 (1)	2 (0)	1 (0)	0	1 (1)
**Other**						
Any level	54 (8)	46 (7)	24 (4)	16 (3)	6 (3)	6 (7)
Short minor	13 (2)	8 (1)	6 (1)	0	2 (1)	2 (2)
Long minor	5 (1)	7 (1)	3 (1)	3 (1)	0	0
Short moderate	12 (2)	13 (2)	2 (1)	7 (1)	1 (1)	3 (4)
Long moderate	24 (3)	18 (3)	13 (2)	6 (1)	3 (1)	1 (1)
**Nausea**						
Any level	31 (4)	27 (4)	16 (3)	10 (5)	2 (1)	3 (3)
Short minor	14 (2)	9 (1)	7 (1)	3 (1)	0	0
Long minor	1 (0)	1 (0)	0	1 (1)	0	0
Short moderate	14 (2)	13 (2)	8 (1)	4 (2)	2 (1)	2 (2)
Long moderate	2 (0)	4 (1)	1 (0)	2 (1)	0	1 (1)

^a^Level: Short minor (NRS ≤ 3 and duration <24 h), Long minor (NRS ≤ 3 and duration ≥24 h), Short moderate (NRS > 3 and duration <24 h), Long moderate (NRS > 3 and duration ≥24 h).

Numeric rating scale (NRS) 0–10 (0 = had not bothered them at all and 10 = had bothered them in the worst possible way).

There were no differences between the treatment groups regarding the occurrence of the four levels of adverse events (Table 3). Comparison between women and men showed that women more often experienced short moderate adverse events (OR = 2.19(95% CI:1.52-3.15), and long moderate adverse events (OR = 2.49(95% CI:1.77-3.52)) (Table 4).

**Table 3:** Comparison of different levels of adverse events between treatment arms

	**Adverse events**			
**Treatment arms**	**Short minor**^**a **^**OR (95% CI)**	**Long minor**^**b **^**OR (95% CI)**	**Short moderate**^**c **^**OR (95% CI)**	**Long moderate**^**d **^**OR (95% CI)**
1. MT^e^	Ref.	Ref.	Ref.	Ref.
2. MT excluding spinal manipulation	1.09 (0.83-1.43)	1.37 (0.91-2.08)	0.82 (0.58-1.16)	1.09 (0.79-1.52)
3. MT excluding stretching	1.09 (0.84-1.43)	1.24 (0.82-1.89)	0.97 (0.70-1.37)	1.11 (0.81-1.53)

^a^(NRS ≤3 and duration <24 hours), ^b^(NRS ≤3 and duration ≥24 hours), ^c^(NRS >3 and duration < 24 hours), ^d^(NRS >3 and duration ≥24 hours).

Numeric rating scale (NRS) 0–10 (0 = had not bothered them at all and 10 = had bothered them in the worst possible way).

^e^Manual Therapy (spinal manipulation/mobilization, stretching and massage).

**Table 4:** Comparison of different levels of adverse events between sexes

	**Adverse events**			
**Sexes**	**Short minor**^**a **^**OR (95% CI)**	**Long minor**^**b **^**OR (95% CI)**	**Short moderate**^**c **^**OR (95% CI)**	**Long moderate**^**d **^**OR (95% CI)**
Men	Ref.	Ref.	Ref.	Ref.
Women	1.05	0.96	2.19	2.49
	(0.82–1.33)	(0.67-1.39)	(1.52-3.15)	(1.77-3.52)

^a^(NRS ≤ 3 and duration < 24 hours), ^b^(NRS ≤ 3 and duration ≥ 24 hours), ^c^(NRS > 3 and duration <24 hours), ^d^(NRS > 3 and duration ≥ 24 hours).

Numeric rating scale (NRS) 0–10 (0 = had not bothered them at all and 10 = had bothered them in the worst possible way).

Table 5 describes the number and proportion of patients who have experienced any kind of adverse events, regardless of severity and duration, after every visit (37%), after any of the visits (51%) or had no adverse events after any of the visits (13%), in the sub-cohort of patients who had at least three treatments (n = 556).

**Table 5:** Patients who experienced at least one adverse event, regardless of duration and severity (level) after three visits (n = 556)

**Adverse events**	**MT**^**a **^**(n = 165)**	**MT excluding spinal manipulation (n = 193)**	**MT excluding stretching (n = 198)**	**Total**
	**n**	**n**	**n**	**n**
	**(%)**	**(%)**	**(%)**	**(%)**
After every visit	63	69	71	203
	(38)	(36)	(36)	(37)
After any visit	82	99	101	282
	(50)	(51)	(51)	(51)
None	20	25	26	71
	(12)	(13)	(13)	(13)

^a^Manual Therapy (spinal manipulation/mobilization, stretching and massage).

Table 6 illustrates the number and proportion of patients who experienced at least one adverse event after each visit. The results are presented per level of adverse event.

**Table 6:** Patients experiencing at least one adverse event after each visit

**Visit number**	**1.**	**2.**	**3.**	**4.**	**5.**	**6.**
	**n = 767**	**n = 685**	**n = 556**	**n = 389**	**n = 211**	**n = 84**
	**(%)**	**(%)**	**(%)**	**(%)**	**(%)**	**(%)**
Adverse event						
Short minor^a^	270	218	147	89	55	32
	(35)	(32)	(26)	(23)	(26)	(38)
Long minor^b^	34	110	64	37	27	12
	(4)	(16)	(12)	(10)	(13)	(14)
Short moderate^c^	135	147	87	57	24	10
	(18)	(21)	(17)	(15)	(11)	(12)
Long moderate^d^	121	117	80	44	18	7
	(16)	(17)	(14)	(11)	(9)	(8)

^a^(NRS ≤3 and duration <24 hours), ^b^(NRS ≤3 and duration ≥24 hours), ^c^(NRS >3 and duration <24 hours), ^c^(NRS >3 and duration <24 hours), ^d^(NRS >3 and duration ≥24 hours).

Numeric rating scale (NRS) 0–10 (0 = had not bothered them at all and 10 = had bothered them in the worst possible way).

## Discussion

This study is based on a RCT of patients seeking care for neck and/or back pain at the educational clinic at the Scandinavian College of Naprapathic Manual Medicine in Stockholm, Sweden. The primary aim of the present study was to report on adverse events after manual therapy and especially to explore if the occurrence of these events varied between different combinations of treatment techniques used. In addition, women and men were compared regarding the occurrence of adverse events.

Our results showed that minor or moderate adverse events were common. Of patients who had at least three treatment sessions, 37% had an event after every visit, 51% had an event after some of the visits and 13% reported no events. Similar results have been found by others [7,8]. Previous studies have also suggested that the severity and duration of adverse events after manual therapy were mild and transient [7,16,28] similar to our results. The most frequent level of adverse event in this study was short minor lasting less than 24 hours and was rated less or equal to three on the numeric rating scale regarding severity. No serious adverse events were reported in this study.

We found no differences in the occurrence of adverse events at any level between the treatment arms. These findings are in line with the results of the previous randomized trials by Leaver et al. and Hondras et al. [29,30]. However, the pain location (neck or lower back) might be of importance. Others have found differences in occurrence of adverse events between spinal manipulation and mobilization in the upper cervical area [31]. Cagnie et al. found that spinal manipulation of the neck was associated with more adverse events compared to those after lumbar manipulation [16]. In our study, more than one third of the patients were treated for low back pain, which partly may explain why we found no differences across the treatment groups.

Our findings show that the most common adverse events were soreness in muscles, increased pain, stiffness and tiredness, which support results from previous studies [17,30]. Our results show further that the occurrence of adverse events is more common early in the treatment series.

Comparison between men and women showed that there were no differences concerning reported minor adverse events. However, men reported fewer short and long moderate adverse events.

### Strengths

The main strength of this study is the design. Randomization of the patients leads to high internal validity by lowering the possibility of confounded results. A comparison of the characteristics between the three arms in Table 1 shows that the groups are equal regarding potential confounders. Furthermore, we have a large study (n = 767), a 97% participation rate and a low attrition rate, 6% with similar attrition rate in each treatment arm.

The fact that the treatments were carried out by many trained therapists (260 therapists) enables generalizing of the results to the therapy rather than to a few therapists.

A trained research assistant not involved in the treatment analyses or in the writing of the manuscript was responsible for the randomization, the data collection, the data input and data quality management. The data analyses were done by one of the authors (KP) who had no influence on the randomization and data collection process.

Most of the published studies regarding spinal manipulation/mobilization have compared the treatment effect, and adverse events have been presented as secondary findings. In this study we have focus on adverse events only, which enables us to present detailed results, which are of relevance and importance to patients and manual therapists.

The patients reported adverse events by filling in a questionnaire when they showed up at the return visit. The treatment interval was approximately one week, and we have no reason to believe that there is any recall bias, or that any potential recall bias should differ between the treatment groups.

### Limitations

The fact that the patients were treated by students, trained for this trial, with short clinical experience in manual treatment techniques may raise the question whether our results are applicable to treatments given by experienced therapists. All therapist students in this study have been treating patients, under the supervision of experienced naprapaths, regularly (two days/week) during three years before the study inception, and they had passed all practical and theoretical examinations required at this level of education. However, there might still be differences in their skills which may have an impact on the external validity.

Another concern may be that the patients visiting an educational clinic differ from patients visiting registered naprapaths in Stockholm, Sweden. To investigate this, we performed a study. The results of that study suggested that the patients in this study were younger (35.0 [SD 12.1] years) compared to patients visiting other clinics in Stockholm (42.0 [10.7] years). The mean number of prior naprapath visits in the present study was 1,86 (SD = 0,85) compared to 2,51 (SD = 0.68) for those visiting other clinics. Other parameters like levels of education, physical activity, pain and disability scores were equal in the two populations (unpublished data by Joakim Ahlgren).

The pragmatic nature of the interventions and the limited experience of the therapists may constitute a risk that the therapists did not follow the study protocol and the result of the randomization and therefore the treatments were not sufficiently differentiated. This could be a reason why we did not find any differences between the treatment arms. The therapists were told to use spinal manipulation in arm 1 and 3, and stretching in arm 1 and 2, if there were indications for that, in regular meetings during the period of inclusion. In an attempt to assure that treatments were carried out according to the randomization and the protocol, we randomly selected and read 6% of the patient records. This scan showed that the therapists had performed the treatments according to the protocol.

To avoid differences in expectations regarding the study arms the therapist was encouraged not to tell the patient the result of the randomization. However, due to the nature of the intervention the patients who are used to manual treatments may be affected negatively or positively when they realize the consequence of the randomization.

The therapist was not blinded for the adverse events experienced by patients after previous visit. This may constitute a potential risk that patient over/under report the duration and/or severity of adverse events. However, we don’t consider this potential misclassification of the outcome to differ between the treatment arms.

There may be a concern regarding that the symptoms reported as adverse events potentially could originate from other sources than from the current treatment. This potential bias should be a non-differential misclassification of the outcome and have a dilutive effect on the results.

A potential bias related to difficulties to recall details about adverse events is if too long time has passed between the treatment session and the measures of adverse events. The proportion of patients filling in the adverse event questionnaire within two weeks was 64% and within one month 21%, indicating that this may be a bias, but we have no reason to believe that the recall differs between treatment arms.

Soreness in muscles was the most common adverse event. This might not be unexpected since the treatment affects an injured area of the body which probably will cause discomfort to the patients afterwards. It possible that soreness in muscles should not be considered an adverse event but rather a normal reaction due to the treatment, as far as the reaction is mild and transient.

Finally, although the questionnaire used in this study, was guided by questions used in previous studies, no formal validation or reliability of the questionnaires has been made. A pilot study was conducted prior to this RCT with the aim to test the questionnaire and the feasibility of the current study. The patients (n = 45) from the pilot study were all included in the RCT since no changes were made to the questionnaires, the methodology, the randomization allocation or content of treatments after the pilot study.

We consider that the results from this study are clinically applicable and important both for the patients and for manual therapist.

## Conclusion

Adverse events after manual therapy are common and transient. Excluding spinal manipulation or stretching do not affect the occurrence of adverse events. The most common adverse event is soreness in the muscles. Women report more adverse events than men.

## Competing interest

All authors have completed the ICMJE uniform disclosure at http://www.icmje.org/coi_disclosure.pdf and declare: financial support for the submitted work from the Swedish Naprapathic Association (SNA) and the Scandinavian College of Naprapathic Manual Medicine (SCNMM). KP doctoral student position is financed by the SNA and the SCNMM. ES has a part time position at the SCNMM and the part time position at Karolinska Institutet is financed by grants from the SCNMM and from the SNA. LWH does consultancy for SCNMM, MN declare no conflict of interest, MA has a part time position at the SCNMM and JL does consultancy for SCNMM.

## Authors’ contributions

All the authors were involved in the design, procedure and data analyses of the study, and the requirements for authorship have been met. KP was responsible for writing the paper, but all the other authors have participated throughout the writing process and have read and approved the final version.

## Pre-publication history

The pre-publication history for this paper can be accessed here:

http://www.biomedcentral.com/1471-2474/15/77/prepub

## References

[B1] Demyttenaere K Bruffaerts R Lee S Posada-Villa J Kovess V Angermeyer MC Levinson D de Girolamo G Nakane H Mneimneh Z Lara C de Graaf R Scott KM Gureje O Stein DJ Haro JM Bromet EJ Kessler RC Alonso J Von Korff M Mental disorders among persons with chronic back or neck pain: results from the World Mental Health Surveys Pain 2007 129 3 332 342 10.1016/j.pain.2007.01.022 17350169

[B2] Manchikanti L Singh V Datta S Cohen SP Hirsch JA Comprehensive review of epidemiology, scope, and impact of spinal pain Pain Physician 2009 12 4 E35 70 19668291

[B3] Koes BW van Tulder MW Ostelo R Kim Burton A Waddell G Clinical guidelines for the management of low back pain in primary care: an international comparison Spine (Phila Pa 1976) 2001 26 22 2504 2513 discussion 2513–2504 10.1097/00007632-200111150-00022 11707719

[B4] Paciaroni M Bogousslavsky J Cerebrovascular complications of neck manipulation Eur Neurol 2009 61 2 112 118 10.1159/000180314 19065058

[B5] Ernst E Adverse effects of spinal manipulation: a systematic review J R Soc Med 2007 100 7 330 338 10.1258/jrsm.100.7.330 17606755 PMC1905885

[B6] Hurwitz EL Morgenstern H Vassilaki M Chiang LM Adverse reactions to chiropractic treatment and their effects on satisfaction and clinical outcomes among patients enrolled in the UCLA Neck Pain Study J Manipulative Physiol Ther 2004 27 1 16 25 10.1016/j.jmpt.2003.11.002 14739870

[B7] Eriksen K Rochester RP Hurwitz EL Symptomatic reactions, clinical outcomes and patient satisfaction associated with upper cervical chiropractic care: a prospective, multicenter, cohort study BMC Musculoskelet Disord 2011 12 219 10.1186/1471-2474-12-219 21974915 PMC3204272

[B8] Carnes D Mars TS Mullinger B Froud R Underwood M Adverse events and manual therapy: a systematic review Man Ther 2010 15 4 355 363 10.1016/j.math.2009.12.006 20097115

[B9] Cassidy JD Boyle E Cote P He Y Hogg-Johnson S Silver FL Bondy SJ Risk of vertebrobasilar stroke and chiropractic care: results of a population-based case–control and case-crossover study Spine (Phila Pa 1976) 2008 33 4 Suppl S176 S183 18204390 10.1097/BRS.0b013e3181644600

[B10] Gouveia LO Castanho P Ferreira JJ Safety of chiropractic interventions: a systematic review Spine (Phila Pa 1976) 2009 34 11 E405 413 10.1097/BRS.0b013e3181a16d63 19444054

[B11] Choi S Boyle E Cote P Cassidy JD A population-based case-series of Ontario patients who develop a vertebrobasilar artery stroke after seeing a chiropractor J Manipulative Physiol Ther 2011 34 1 15 22 10.1016/j.jmpt.2010.11.001 21237403

[B12] Hurwitz EL Morgenstern H Harber P Kominski GF Yu F Adams AH A randomized trial of chiropractic manipulation and mobilization for patients with neck pain: clinical outcomes from the UCLA neck-pain study Am J Public Health 2002 92 10 1634 1641 10.2105/AJPH.92.10.1634 12356613 PMC1447299

[B13] Gross AR Hoving JL Haines TA Goldsmith CH Kay T Aker P Bronfort G A Cochrane review of manipulation and mobilization for mechanical neck disorders Spine (Phila Pa 1976) 2004 29 14 1541 1548 10.1097/01.BRS.0000131218.35875.ED 15247576

[B14] Hakkinen A Salo P Tarvainen U Wiren K Ylinen J Effect of manual therapy and stretching on neck muscle strength and mobility in chronic neck pain J Rehabil Med 2007 39 7 575 579 10.2340/16501977-0094 17724558

[B15] da Costa BR Vieira ER Stretching to reduce work-related musculoskeletal disorders: a systematic review J Rehabil Med 2008 40 5 321 328 10.2340/16501977-0204 18461255

[B16] Cagnie B Vinck E Beernaert A Cambier D How common are side effects of spinal manipulation and can these side effects be predicted? Man Ther 2004 9 3 151 156 10.1016/j.math.2004.03.001 15245709

[B17] Skillgate E Vingard E Alfredsson L Naprapathic manual therapy or evidence-based care for back and neck pain: a randomized, controlled trial Clin J Pain 2007 23 5 431 439 10.1097/AJP.0b013e31805593d8 17515742

[B18] Key W Författningshandbok för personal inom hälso- och sjukvården 2004 Stockholm: Liber 64 68

[B19] Skillgate E Vingard E Josephson M Theorell T Alfredsson L Life events and the risk of low back and neck/shoulder pain of the kind people are seeking care for: results from the MUSIC-Norrtalje case–control study J Epidemiol Community Health 2007 61 4 356 361 10.1136/jech.2006.049411 17372298 PMC2652947

[B20] Ware J Jr Kosinski M Keller SD A 12-Item Short-Form Health Survey: construction of scales and preliminary tests of reliability and validity Med Care 1996 34 3 220 233 10.1097/00005650-199603000-00003 8628042

[B21] von Korff M Ormel J Keefe FJ Dworkin SF Grading the severity of chronic pain Pain 1992 50 2 133 149 10.1016/0304-3959(92)90154-4 1408309

[B22] Senstad O Leboeuf-Yde C Borchgrevink C Frequency and characteristics of side effects of spinal manipulative therapy Spine (Phila Pa 1976) 1997 22 4 435 440 discussion 440–431 10.1097/00007632-199702150-00017 9055373

[B23] Barrett AJ Breen AC Adverse effects of spinal manipulation J R Soc Med 2000 93 5 258 259 10884771 10.1177/014107680009300511 PMC1298004

[B24] Rubinstein SM Leboeuf-Yde C Knol DL de Koekkoek TE Pfeifle CE van Tulder MW The benefits outweigh the risks for patients undergoing chiropractic care for neck pain: a prospective, multicenter, cohort study J Manipulative Physiol Ther 2007 30 6 408 418 10.1016/j.jmpt.2007.04.013 17693331

[B25] Hoogendoorn WE Bongers PM de Vet HC Twisk JW van Mechelen W Bouter LM Comparison of two different approaches for the analysis of data from a prospective cohort study: an application to work related risk factors for low back pain Occup Environ Med 2002 59 7 459 465 10.1136/oem.59.7.459 12107294 PMC1740320

[B26] Zeger SL Liang KY Longitudinal data analysis for discrete and continuous outcomes Biometrics 1986 42 1 121 130 10.2307/2531248 3719049

[B27] StataCorp Stata Statistical Software: Release 12 2011 College Station, TX: StataCorp LP

[B28] Bronfort G Evans R Anderson AV Svendsen KH Bracha Y Grimm RH Spinal manipulation, medication, or home exercise with advice for acute and subacute neck pain: a randomized trial Ann Intern Med 2012 156 1 Pt 1 1 10 22213489 10.7326/0003-4819-156-1-201201030-00002

[B29] Leaver AM Maher CG Herbert RD Latimer J McAuley JH Jull G Refshauge KM A randomized controlled trial comparing manipulation with mobilization for recent onset neck pain Arch Phys Med Rehabil 2010 91 9 1313 1318 10.1016/j.apmr.2010.06.006 20801246

[B30] Hondras MA Long CR Cao Y Rowell RM Meeker WC A randomized controlled trial comparing 2 types of spinal manipulation and minimal conservative medical care for adults 55 years and older with subacute or chronic low back pain J Manipulative Physiol Ther 2009 32 5 330 343 10.1016/j.jmpt.2009.04.012 19539115

[B31] Hurwitz EL Morgenstern H Vassilaki M Chiang LM Frequency and clinical predictors of adverse reactions to chiropractic care in the UCLA neck pain study Spine (Phila Pa 1976) 2005 30 13 1477 1484 10.1097/01.brs.0000167821.39373.c1 15990659

